# Born blonde: a recessive loss‐of‐function mutation in the melanocortin 1 receptor is associated with cream coat coloration in Antarctic fur seals

**DOI:** 10.1002/ece3.2290

**Published:** 2016-07-22

**Authors:** Lucy Peters, Emily Humble, Nicole Kröcker, Birgit Fuchs, Jaume Forcada, Joseph I. Hoffman

**Affiliations:** ^1^ Department of Animal BehaviourUniversity of BielefeldPostfach 10013133501BielefeldGermany; ^2^ College of Medical, Veterinary & Life SciencesUniversity of GlasgowGraham Kerr BuildingGlasgowG12 8QQUK; ^3^ British Antarctic SurveyHigh Cross, Madingley RoadCambridgeCB3 OETUK

**Keywords:** Antarctic fur seal (*Arctocephalus gazella*), candidate gene, coat coloration, inbreeding, melanocortin 1 receptor

## Abstract

Although the genetic basis of color variation has been extensively studied in humans and domestic animals, the genetic polymorphisms responsible for different color morphs remain to be elucidated in many wild vertebrate species. For example, hypopigmentation has been observed in numerous marine mammal species but the underlying mutations have not been identified. A particularly compelling candidate gene for explaining color polymorphism is the melanocortin 1 receptor (*MC1R*), which plays a key role in the regulation of pigment production. We therefore used Antarctic fur seals (*Arctocephalus gazella*) as a highly tractable marine mammal system with which to test for an association between nucleotide variation at the *MC1R* and melanin‐based coat color phenotypes. By sequencing 70 wild‐type individuals with dark‐colored coats and 26 hypopigmented individuals with cream‐colored coats, we identified a nonsynonymous mutation that results in the substitution of serine with phenylalanine at an evolutionarily highly conserved structural domain. All of the hypopigmented individuals were homozygous for the allele coding for phenylalanine, consistent with a recessive loss‐of‐function allele. In order to test for cryptic population structure, which can generate artefactual associations, and to evaluate whether homozygosity at the *MC1R* could be indicative of low genome‐wide heterozygosity, we also genotyped all of the individuals at 50 polymorphic microsatellite loci. We were unable to detect any population structure and also found that wild‐type and hypopigmented individuals did not differ significantly in their standardized multilocus heterozygosity. Such a lack of association implies that hypopigmented individuals are unlikely to suffer disproportionately from inbreeding depression, and hence, we have no reason to believe that they are at a selective disadvantage in the wider population.

## Introduction

The sheer diversity of vertebrate pigmentation has long fascinated animal breeders and evolutionary biologists. Studies of the mechanistic basis of color variation have identified a number of key genes required for melanocyte development, migration, and regulation (Kerns et al. 2003) while also providing valuable insights into sexual selection (Andersson 1994), adaptive evolution (Nachman et al. 2003), gene action (Kaelin and Barsh 2013), and the inheritance of genetic traits in general (Kaelin and Barsh, 2013). Intriguingly, coloration has also been linked to fitness variation in several species (Kruger et al. 2001; Drogemuueller et al. 2007; Dessinioti et al. 2011), despite many of the genes known to be involved having no obvious pleiotropic effects (Mundy 2005; Bellone 2010). This has stimulated ongoing research into the underlying mechanisms at the level of the genome (e.g., Küpper et al. 2016; Lamichhaney et al. 2016).

One of several possible explanations for fitness differences among color morphs is heterozygote advantage. For example, in the common buzzard (*Buteo buteo*), three color morphs have been described that differ in their levels of plumage melanisation, termed “light,” “intermediate,” and “dark”. Plumage morph follows Mendelian expectations for a single locus with two alleles (Kruger et al. 2001), and the heterozygous intermediate morph is on average longer lived and has the greatest lifetime reproductive success (Chakarov et al. 2008). A recent study that sequenced the genome of the captive albino gorilla *Snowflake* also points toward a possible link between coloration and inbreeding (Prado‐Martinez et al. 2013). This could arise because recessive loss‐of‐function alleles are more likely to be expressed in individuals whose genome‐wide heterozygosity is reduced as a result of close inbreeding.

The melanocortin 1 receptor (*MC1R*) is a classical candidate gene for explaining color polymorphism (Barsh 1996). This gene encodes a G‐protein‐coupled receptor that is expressed in the membrane of melanocytes and whose activity is controlled by two ligands, the *α*‐melanocortin‐stimulating hormone (*α*‐MSH) and agouti. The binding of the former activates the MC1R and stimulates synthesis of the black to brown eumelanin, while antagonism of *α*‐MSH by agouti signaling protein suppresses MC1R activity and results in the production of yellow to red pheomelanin (Gantz and Fong 2003). Mutations in the *MC1R* gene have been shown to induce a change in pigmentation in humans (Schioth et al. 1999) as well as in domestic animals including pigs, cows, chickens, horses, and dogs (Klungland et al. 1995; Marklund et al. 1996; Takeuchi et al. 1996; Kijas et al. 1998; Newton et al. 2000; Oguro‐Okano et al. 2011). Genetic variation at the *MC1R* has also been associated with melanic coat color variation in natural populations of around 20 species belonging to three mammalian orders, five avian orders, and lizards (Hoekstra 2006). However, the vast majority of wild species have not yet been studied (although see Theron et al. 2001; Eizirik et al. 2003; Nachman et al. 2003) probably due to the difficulty of designing PCR primers in nonmodel organisms that lack genomic resources.

Marine mammals are a large and diverse vertebrate clade in which anomalous coloration has been widely reported. For example, albinism and leucism have been reported in 22 different cetacean (Fertl et al. 1999, 2004) and seven different pinniped species (Bartholomew and Hubbs 1952; Bried and Haubreux 2000; Acevedo and Aguayo 2008; Bester et al. 2008). However, affected individuals tend to be rare and genetic samples are often very difficult to collect from marine mammals, precluding robust studies of the genetic mechanisms underlying such variation. In marine mammals, it has also been suggested that abnormal coloration could carry costs, potentially including reduced heat absorption in colder waters, poor camouflage from predators, and increased sensitivity to sunlight (Fertl and Rosel 2008). A further possibility is that if hypopigmentation is associated with inbreeding, then affected individuals could be at a fitness disadvantage due to inbreeding depression.

**Figure 1:**
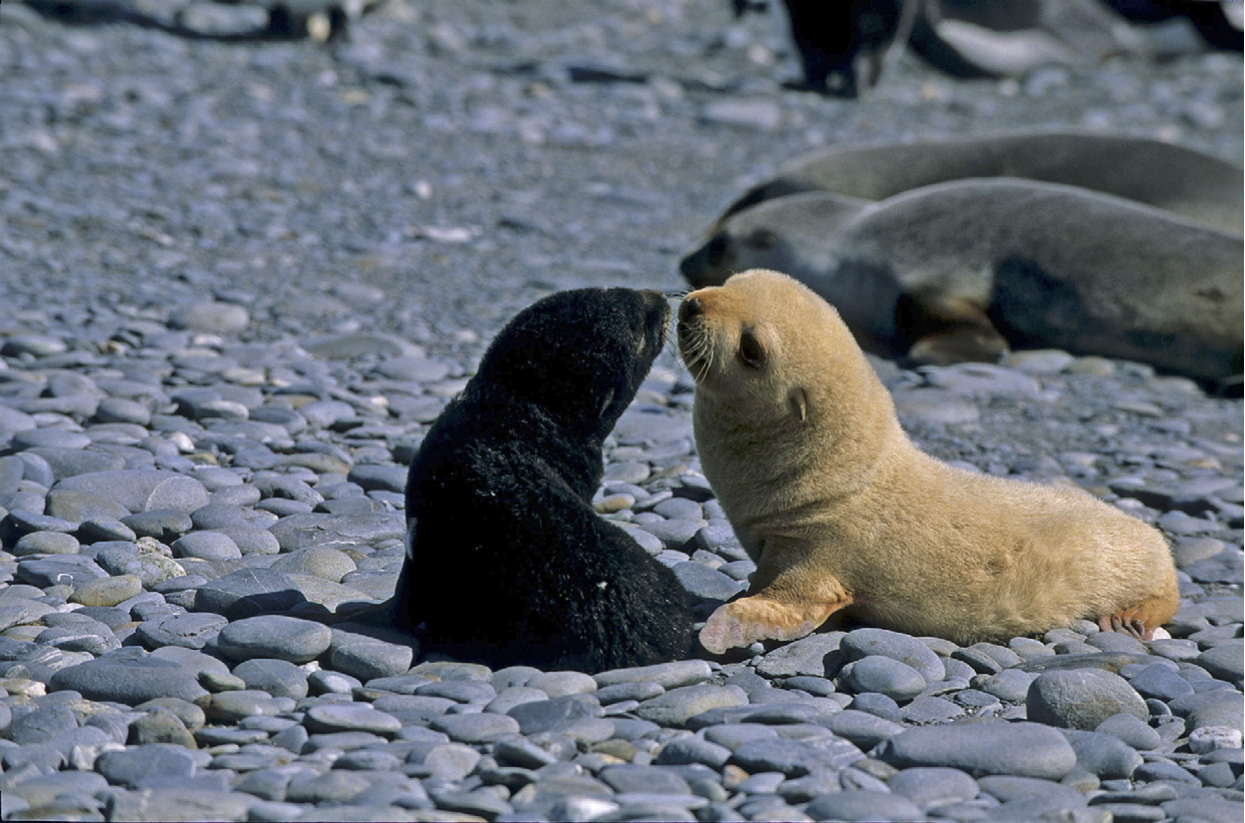
Antarctic fur seal pups at South Georgia, showing the wild‐type (left) and hypopigmented (right) phenotypes. Photograph credit: Oliver Krüger.

A long‐term study of a breeding colony of Antarctic fur seals (*Arctocephalus gazella*) provides a unique opportunity to study the genetic basis of color polymorphism in a marine mammal and to look for a possible link between pigmentation and inbreeding. Whereas most individuals of this species have dark brown fur, a distinctive cream color morph has also been described (de Bruyn et al. 2007; Wege et al. 2015) (see (Fig. 1). The latter has been attributed to leucism (Bonner 1968; de Bruyn et al. 2007; Acevedo and Aguayo 2008; Bester et al. 2008; Wege et al. 2015), a form of hypopigmentation that is distinct from albinism as the eyes and body extremities remain normally colored (Wege et al. 2015). However, leucism is caused by defects in pigment cell differentiation, whereas the form of hypopigmentation observed in fur seals is characterized by a reduced amount of melanin production which leads to a cream (pheomelanic) phenotype, as also observed in yellow Labradors (Newton et al. 2000). Hypopigmented fur seals have mainly been observed at South Georgia, where around 97% of the extant population currently breeds, as well as in the nearby South Shetland Islands (Bonner 1968; Acevedo et al. 2009). Here, they are thought to occur at a frequency of between one in 600 and one in 1700 individuals (Bonner 1968; Aguayo 1978). More recently, hypopigmented individuals have also been sighted further afield at Marion Island in the sub‐Antarctic Indian Ocean (de Bruyn et al. 2007; Wege et al. 2015), where their presence has been interpreted as providing evidence for recent population expansion probably radiating from South Georgia (Wege et al. 2015).

Antarctic fur seals are relatively convenient for studying the molecular genetics of pigmentation for three main reasons. First, they aggregate ashore in large numbers during the breeding season, allowing sufficient numbers of hypopigmented individuals to be sampled for genetic analysis despite their rarity. Second, recent observations of a cream‐colored pup born to a wild‐type female and a cream‐colored female nursing a wild‐type pup (Wege et al. 2015) are suggestive of a simple Mendelian trait with a recessive mode of inheritance. Third, the recent publication of a draft Antarctic fur seal genome sequence (Humble et al. 2016) allows the design of species‐specific PCR primers to amplify the entire *MC1R* locus.

Finally, heterozygosity measured at a panel of nine microsatellites correlates positively with virtually every fitness component that it has been possible to measure in Antarctic fur seals ranging from body size, territory holding ability, and reproductive success in males to recruitment and breeding success in females (Hoffman et al. 2004, 2010; Forcada and Hoffman 2014). Consequently, if hypopigmented individuals are more inbred, as appears to be the case for the albino gorilla *Snowflake* (Prado‐Martinez et al. 2013), then they could be at a selective disadvantage due to inbreeding depression, which potentially could contribute toward their low frequency in the wider population.

Here, we sought to identify the genetic polymorphism responsible for hypopigmentation in Antarctic fur seals by sequencing the complete *MC1R* locus in 70 wild‐type and 26 cream‐colored individuals, comprising mainly pups but also several adults including five mothers of hypopigmented pups. These individuals were also genotyped at 50 highly polymorphic microsatellites, allowing us to test the hypothesis that hypopigmentation provides a phenotypic marker of low genome‐wide heterozygosity, or inbreeding.

## Materials and Methods

### Sample collection and DNA extraction

Skin samples were collected from Antarctic fur seals at Bird Island, South Georgia (54°00′S, 38°02′W). The majority of samples (*n *=* *96) were collected from pups (66 wild‐type, and 22 cream‐colored), whereas the remaining samples were obtained from five mothers of cream‐colored pups (one of which was also cream‐colored) and three additional adult hypopigmented individuals, two females and one male (Table S1). All of the animals were captured and restrained on land using standard methodology (Gentry and Holt 1982) that forms part of annual routine procedures of the long‐term ecosystem studies of the British Antarctic Survey. Skin samples were collected from the interdigital margin of the foreflipper using piglet ear notching pliers (Majluf and Goebel 1992). Skin samples were stored individually in the preservative buffer 20% dimethyl sulphoxide saturated with salt (Amos and Hoelzel 1991) and stored at −20°C. Total genomic DNA was extracted from the skin samples using a standard phenol‐chloroform protocol (Sambrook et al. 1989).

### *MC1R* sequencing

The full length (954 bp) *MC1R* receptor sequence of the dog (*Canis lupus familiaris*) was downloaded from Genbank (accession number NM_001014282 XM_546772, chromosome 5) and blasted against the Antarctic fur seal reference genome (Humble et al. 2016) at an *e*‐value threshold of 1*e* ^−12^. The corresponding fur seal sequence and its 500‐bp flanking regions was extracted from the genome using the bedtools command *getfasta* (Quinlan and Hall 2010). This sequence was then used to design three primer pairs using the program Primer3 (Untergasser et al. 2007) (5′‐ ctggagatgggtgcttcttc ‐3′/5′‐ tctttgtagccatgctggtg ‐3′, 5′‐tgaaaggtgcaggaagaagg‐3′/5′‐ atcgccaagaaccgcaac‐3′, and 5′‐ ccaggcagcagatgaagtaa ‐3′/5′‐ ggaagctggggtctcttcag ‐3′) to PCR‐amplify overlapping fragments of length 537, 600 and 394 nucleotides, respectively. Each PCR was conducted in a 10 *μ*L reaction volume containing 100 ng of template DNA, 20 mmol/L Tris–HCl (pH 8.3), 100 mmol/L KCl, 2 mmol/L MgCl_2_, 0.1 mmol/L EDTA, 0.25 mmol/L dNTPs, 0.25 *μ*mol/L of each primer, and 0.5 units of 5PrimeTaq polymerase (VWR). The following PCR profile was used for the first fragment: one cycle of 5 min at 94°C; 30 cycles of 30 sec at 94°C, 60 sec at 63.4°C, and 60 sec at 72°C; and one final cycle of 7 min at 72°C. The PCR profile of the second and third fragment only differed from this protocol in their annealing temperatures, which were 64.6 and 65.2°C, respectively. A total of 10 *μ*L of the resulting PCR product was purified using shrimp alkaline phosphatase and exonuclease I (New England Biolabs: Ipswich, Massachusetts, USA) following the manufacturer's recommended protocol. All fragments were then sequenced in both directions using the Applied Biosystems BigDye^®^ Terminator v3.1 Cycle Sequencing Kit (Thermo Fisher Scientific: Waltham, Massachusetts, USA) and analyzed on an ABI 3730xl capillary sequencer.

### Microsatellite genotyping

All of the samples were genotyped at a panel of 50 polymorphic microsatellite loci. These were individually fluorescently labeled and PCR‐amplified in 12 separate multiplexed reactions using a Type It Kit (Qiagen: Venlo, Netherlands) as detailed in Table 1. The following PCR profile was used for all multiplex reactions except for multiplex one: one cycle of 5 min at 94°C; 28 cycles of 30 sec at 94°C, 90 sec at 60°C, and 30 sec at 72°C, followed by a final cycle of 30 min at 60°C. The PCR profile of multiplex one only differed from this protocol in the annealing temperature used, which was 53°C. PCR products were resolved by electrophoresis on an ABI 3730xl capillary sequencer, and allele sizes were scored automatically using the program GeneMarker version 1.95 (SoftGenetics: State College, Pennsylvania, USA). To ensure high genotype quality, all traces were manually inspected by two observers (L.P and J.I.H) within GeneMarker and any incorrect calls were adjusted accordingly.

**Table 1:** Characteristics of the 50 microsatellite loci used in this study including literature sources, allelic richness and allelic size ranges, observed (*H*_o_) and expected (*H*_e_) heterozygosities and FDR‐corrected *P*‐values for deviation from HWE

Locus	Reference	Fluorescent label used	Multiplex	Number of alleles	Allelic size range (bp)	*P*‐value	*H*_o_	*H*_e_
Pv9	Allen et al. (1995)	FAM	1	10	166–186	0.684	0.753	0.784
Hg6.3	Allen et al. (1995)	FAM	1	12	215–245	0.715	0.924	0.878
Hg8.10	Allen et al. (1995)	VIC	1	4	162–184	0.684	0.355	0.382
PvcA	Coltman et al. (1996)	PET	1	8	137–157	0.831	0.868	0.877
Hg1.3	Gemmell et al. (1997)	VIC	1	11	234–266	0.684	0.767	0.786
Zcwb07	Hoffman et al. (2007)	PET	1	13	178–202	0.684	0.833	0.879
Agaz2	Hoffman (2009)	PET	1	9	220–244	0.946	0.824	0.800
Ag3	Hoffman et al. (2008)	FAM	2	2	147–149	0.255	0.407	0.326
Agaz6	Hoffman (2009)	FAM	2	6	174–180	0.699	0.791	0.687
OrrFCB7	Buchanan et al. (1998)	FAM	2	9	194–210	0.255	0.843	0.865
Ag2	Hoffman et al. (2008)	VIC	2	7	212–236	0.867	0.742	0.770
OrrFCB2	Buchanan et al. (1998)	NED	2	11	108–132	0.684	0.849	0.864
Lw10	Davis et al. (2002)	NED	2	17	100–140	0.273	0.933	0.909
ZcwC01	Hoffman et al. (2007)	PET	2	11	131–161	0.684	0.831	0.873
Agaz5	Hoffman, (2009)	PET	2	3	192–196	0.273	0.440	0.520
ZcwDhB.14	Hernandez‐Velazquez et al. (2005)	PET	2	6	226–258	0.684	0.822	0.761
Ssl301	Huebinger et al. (2007)	FAM	3	14	258–290	0.934	0.900	0.894
Ag7	Hoffman et al. (2008)	VIC	3	7	119–137	0.719	0.778	0.772
Ag10	Hoffman et al. (2008)	VIC	3	4	211–217	0.698	0.385	0.398
ZcwDh4.7	Hernandez‐Velazquez et al. (2005)	VIC	3	13	250–276	0.460	0.876	0.877
ZcwE05	Unpublished	NED	3	9	188–204	0.843	0.846	0.815
Ag1	Hoffman et al. (2008)	PET	3	10	101–121	0.684	0.867	0.874
OrrFCB8	Buchanan et al. (1998)	PET	3	8	180–202	0.684	0.795	0.803
Agt‐47	Hoffman and Nichols (2011)	PET	3	3	237–245	0.684	0.467	0.497
ZcwF07	Hoffman et al. (2007)	FAM	4	8	136–150	0.796	0.859	0.790
ZcwD02	Wolf et al. (2005)	FAM	4	13	202–252	0.684	0.837	0.854
ZcwCgDh1.8	Hernandez‐Velazquez et al. (2005)	HEX	4	7	149–173	0.947	0.795	0.708
ZcCgDh5.8	Hernandez‐Velazquez et al. (2005)	VIC	4	12	320–344	0.947	0.864	0.877
M11a	Hoelzel et al. (1999)	NED	4	16	168–204	0.843	0.923	0.923
ZcwE12	Hoffman et al. (2007)	FAM	5	8	180–194	0.843	0.769	0.780
Hg6.1	Allen et al. (1995)	VIC	5	13	138–174	0.831	0.857	0.857
Lc28	Davis et al. (2002)	PET	5	8	136–156	0.954	0.879	0.841
ZcwC03	Wolf et al. (2005)	ROX	5	14	240–270	0.831	0.811	0.872
ZcwA05	Hoffman et al. (2007)	FAM	6	18	90–136	0.831	0.822	0.907
ZcwB09	Wolf et al. (2005)	VIC	6	12	180–208	0.831	0.835	0.852
ZcwC11	Wolf et al. (2005)	NED	6	13	223–253	0.684	0.879	0.894
ZcwE03	Wolf et al. (2005)	FAM	7	10	215–235	0.867	0.769	0.825
ZcwE04	Hoffman et al. (2007)	VIC	7	12	112–140	0.831	0.882	0.868
Pv11	Goodman (1997)	VIC	7	3	144–162	1.000	0.176	0.165
Agaz10	Hoffman (2009)	NED	7	10	150–168	0.158	0.692	0.755
Agaz3	Hoffman (2009)	PET	7	3	204–212	0.946	0.648	0.645
PvcE	Coltman et al. (1996)	VIC	8	12	100–144	0.684	0.876	0.853
ZcwA12	Hoffman et al. (2007)	VIC	8	17	174–222	0.831	0.868	0.860
Mang36	Sanvito et al. (2013)	ROX	9	3	327–333	0.684	0.110	0.125
962‐1	Unpublished data	FAM	10	4	123–129	0.684	0.593	0.557
554‐6	Unpublished data	FAM	10	2	149–151	1.000	0.157	0.146
101‐26	Unpublished data	HEX	10	6	127–139	0.831	0.820	0.757
928‐4b	Unpublished data	HEX	10	10	160–188	0.684	0.898	0.838
507‐11	Unpublished data	HEX	10	3	202–206	0.684	0.539	0.535
Mang44	Sanvito et al. (2013)	ROX	11	5	147–163	0.158	0.681	0.718

### Sequence analysis

Complete consensus sequences based on forward and reverse reads of the three overlapping PCR fragments were generated for each of the individuals using the program ChromasPro version 1.3.4 (Technelysium: South Brisbane, Queensland, Australia). Heterozygous sites were identified as those with two peaks of roughly equal intensity but around half the intensity of a homozygote. All sequences were then imported into BioEdit version 5.0.6 (Hall 1999) and aligned to the segment of the fur seal reference genome containing the *MC1R* sequence. After translating the nucleotide sequences into amino acid sequences and aligning them to the dog MC1R protein in BioEdit to verify their identity, the protein was further characterized using the InterPro protein family database to predict the presence of important sites and domains (Mitchell et al. 2015) followed by a protein fingerprint scan using PRINTS version 42.0 (Attwood et al. 2012). Nonsynonymous amino acid substitutions were analyzed using the nonsynonymous SNP scoring service provided by the PANTHER database version 10 (Mi et al. 2016), which calculates a substitution position‐specific evolutionary conservation score (subPSEC) based on multiple alignments of related proteins using hidden Markov models (Thomas et al. 2006). After aligning the Antarctic fur seal MC1R protein sequence to equivalent sequences from other vertebrate species (see Table S2), we generated a Bayesian phylogenetic tree in BEAST version 1.8.3 (Drummond et al. 2012). For this, we used the JTT amino acid substitution model, which was selected after applying maximum likelihood estimation of model parameters to our alignment in order to find the best‐fit model as implemented in ProtTest version 3.2 (Darriba et al. 2011). A relaxed clock was used together with a Yule speciation process, and a total of one million Markov chain Monte Carlo (MCMC) steps were implemented with samples taken every 100 steps and a burn‐in period of 10,000.

### Microsatellite data analysis

Genepop (Raymond and Rousset 1995) was used to calculate observed and expected heterozygosities and to test for deviations from Hardy–Weinberg equilibrium (HWE) and for linkage disequilibrium, specifying a dememorization number of 10,000, 1000 batches, and 10,000 iterations per batch. We also conducted a Bayesian cluster analysis of the microsatellite genotype dataset using Structure version 2.3.3 (Pritchard et al. 2000). We ran five independent runs each for *K *=* *1–10 using 1 × 10^6^ MCMC iterations after a burn‐in of 1 × 10^5^, specifying the correlated allele frequencies model and assuming admixture. The most likely *K* was then evaluated using the maximal average value of Ln *P*(*D*), a model‐choice criterion that estimates the posterior probability of the data. Finally, we used the R package *inbreedR* (Stoffel et al. in press) to calculate each individual's standardized multilocus heterozygosity (sMLH), defined as the total number of heterozygous loci in an individual divided by the sum of average observed heterozygosities in the population over the subset of loci successfully typed in the focal individual (Coltman et al. 1999). The dataset was then divided into hypopigmented and wild‐type pups, and a Welch two‐sample *t*‐test was performed to test for a difference in sMLH between the two.

### Ethical note

Fur seal samples were collected as part of the Polar Science for Planet Earth program of the British Antarctic Survey, which has employed consistent sampling protocols since 1994. Sampling was authorized by the Senior Executive and the Environment Officers of the Government of South Georgia and the South Sandwich Islands, and samples were collected under Scientific Research Permits for the British Antarctic Survey field activities on South Georgia. All procedures used were approved by the British Antarctic Survey Animal Welfare and Ethics Review Body (reference number PEA6).

## Results

In order to test for an association between nucleotide polymorphisms of the *MC1R* gene and coat coloration in Antarctic fur seals, we sequenced 66 wild‐type and 22 cream‐colored pups as well as five mothers of cream‐colored pups and three additional adults at the full 954‐bp coding region of the receptor. A total of 79 complete high quality sequences were obtained, from which we identified six variable sites and 13 unique haplotypes (see Table S3). A Bayesian phylogenetic tree of vertebrate MC1R protein sequences (Figure S1) clustered the wild‐type fur seal sequence as a sister taxon to that of the Weddell seal (*Leptonychotes weddellii*) nested within the Caniformia clade, which also included the Kermode bear (*Ursus americanus kermodei*) and the European mink (*Mustela lutreola*) as well as the domestic dog (*Canis lupus familiaris*).

### Test of association between *MC1R* genotype and coat coloration

**Figure 2:**
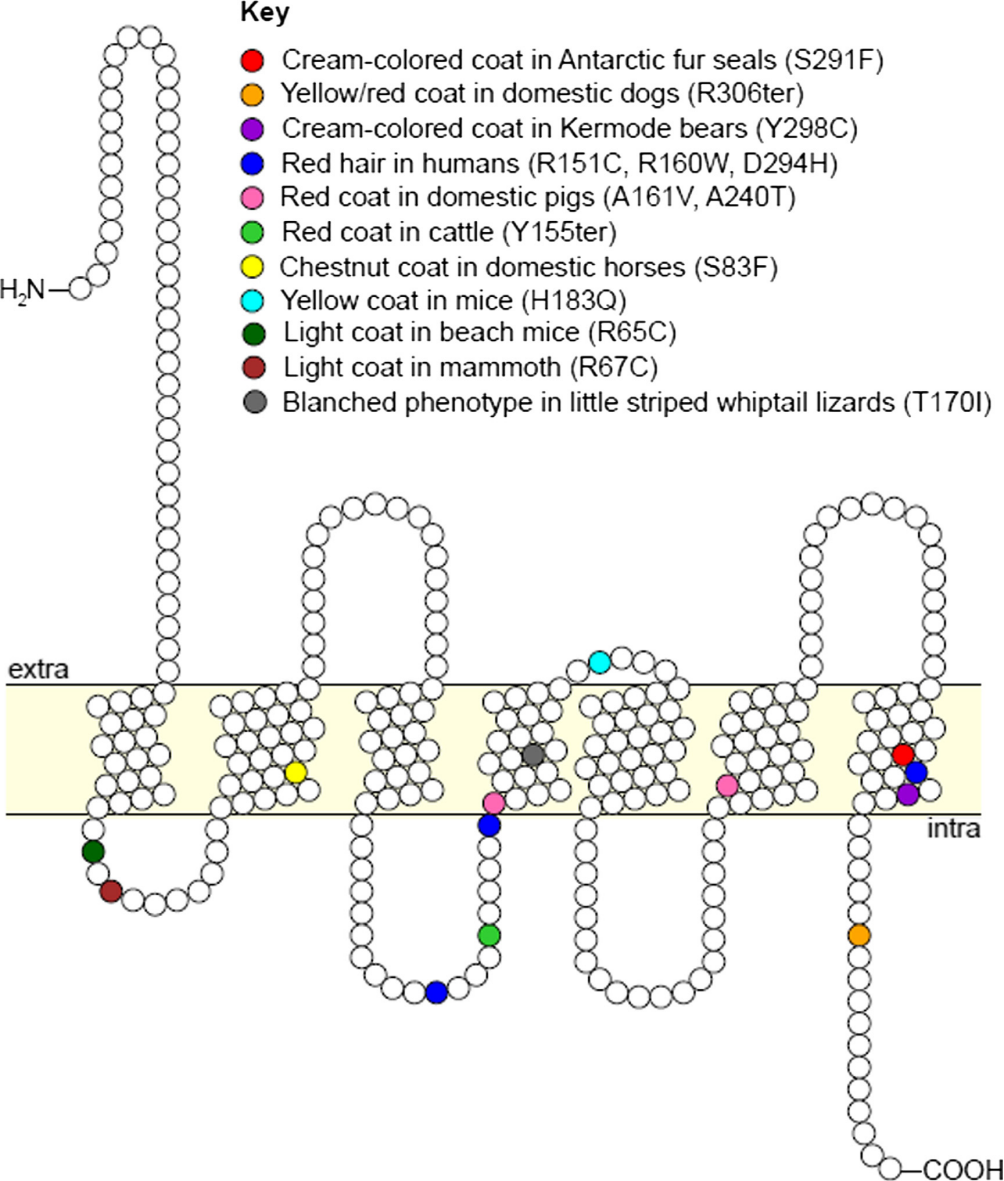
Two‐dimensional representation of the *MC1R* membrane protein showing recessive loss‐of‐function sequence variants associated with coloration in various vertebrate species. See Table S5, for the literature source.

To test for an association between variable sites of the *MC1R* sequence and coat coloration, Fisher's exact tests were performed for each of the variable positions. All six of the variable sites differed significantly between wild‐type and hypopigmented individuals at *P *<* *0.0001 (Table S4). However, only the polymorphism at position 872 in the *MC1R* sequence was perfectly associated with phenotypic variation. This polymorphism was also the only one of the six variable sites that was nonsynonymous. The change from a C to a T at this position resulted in the substitution of serine (codon TCC) with phenylalanine (codon TTC) at position 291 of the amino acid sequence (Fig. 2). All of the hypopigmented pups were homozygous for this mutation. In addition, all of the known mothers of hypopigmented pups were carriers of the variant, as were six of the wild‐type pups.

### Characterization of S291F substitution in MC1R protein sequence

The substitution of serine with phenylalanine at position 291 in the amino acid sequence is a nonconservative one as the former is polar and thus hydrophilic, while the latter is a hydrophobic amino acid. Annotation of the Antarctic fur seal MC1R protein sequence using the InterPro database identified the site of the S291F substitution to be part of the GPCRRHODOPSN motif, a 7‐element identifier of the rhodopsin‐like superfamily, which includes G‐protein‐coupled receptors such as the MC1R. Further investigation of this motif using PRINTS version 42.0 identified the S291F substitution site to be located at the 12th position within the 7th element of the GPCRRHODOPSN fingerprint, which is known to be structurally important with serine being a highly conserved amino acid at this site (Attwood and Findlay 1994).

To further explore whether the S291F substitution is likely to impair protein function, we used PANTHER to calculate a substitution position‐specific evolutionary conservation score based on multiple alignments of related proteins using hidden Markov models (Thomas et al. 2006). The resulting subPSEC score was −3.97, which exceeds the cut‐off value of −3 proposed for identifying mutations that impair protein function (Thomas et al. 2003). The corresponding probability of the variant adversely affecting protein function (*P*_deleterious_) was high at 0.72.

### Microsatellite analyses

We successfully genotyped 85 of the individuals for 50 polymorphic microsatellites. None of these loci deviated significantly from HWE after table‐wide FDR correction, and levels of variability were relatively high, with each locus carrying on average nine alleles (Table 1). As cryptic population structure can generate artefactual associations between *MC1R* genotype and phenotype (Hoffman et al. 2014), we conducted a precautionary Bayesian cluster analysis of the microsatellite dataset using Structure (Pritchard et al. 2000). This program uses a maximum likelihood approach to determine the most likely number of genetically distinct clusters in a sample (*K*) by subdividing the dataset in a way that maximizes HWE and linkage equilibrium within the resulting clusters. The highest average log likelihood value was associated with *K *=* *1 (Figure S2), indicating a lack of population structure.

Finally, we calculated each individual's sMLH based on the 50 microsatellites. No significant difference was found in the sMLH of wild‐type and hypopigmented pups (*t *=* *1.280, *P *=* *0.211), suggesting that homozygosity at the *MC1R* locus is not associated with reduced genome‐wide heterozygosity and hence does not provide a marker of inbreeding.

## Discussion

We tested whether nucleotide variation at the *MC1R* is associated with melanin‐based coat color phenotypes in a model marine mammal species, the Antarctic fur seal. Several lines of evidence point toward a nonsynonymous mutation being responsible, with all of the cream‐colored animals being homozygous for what appears to be a loss‐of‐function allele. We also found no association between multilocus heterozygosity and pelage coloration, suggesting that homozygosity at the color gene does not provide a marker of inbreeding. Such a lack of association implies that hypopigmented individuals are not more likely than wild‐type animals to suffer from inbreeding depression.

We found six polymorphic nucleotide sites within the *MC1R* that were significantly associated with coat coloration. However, only the mutation at position 872 in the fur seal *MC1R* sequence was perfectly associated with the phenotype. The replacement of C with T at this nucleotide position leads to the nonconservative substitution of serine with phenylalanine at site 291 in the amino acid sequence. The allele coding for phenylalanine appears to be recessive to the wild‐type allele as all of the cream‐colored pups were homozygous for this particular allele. Moreover, all four wild‐type mothers of hypopigmented pups were heterozygous, implying that they must have conceived to males who were also carriers of the recessive allele.

Further characterization of the Ser‐to‐Phe replacement provides additional evidence for a loss‐of‐function mutation. Based on domain annotation using the InterPro database, the S291F substitution site is located within the seven‐element GPCRRHODOPSN motif, which functions as an identifier for G‐protein‐coupled receptors such as the MC1R (Attwood and Findlay 1994; Rosenbaum et al. 2009). Protein fingerprint scanning with PRINTS determined that position 291 of the fur seal MC1R amino acid sequence corresponds to position 12 in the seventh element of the GPCRRHODOPSN fingerprint. This particular site has previously been identified to be structurally important, and moreover, serine is highly conserved at this position (Attwood and Findlay 1994). A replacement of serine at this position in the amino acid sequence is therefore likely to affect protein function.

This prediction is further supported by our PANTHER analysis, which suggests that the S291F substitution leads to impaired functioning of the melanocortin receptor based on an alignment of evolutionary related proteins. The subPSEC score of −3.97 falls well below the cut‐off of −3 for damaging mutations (Thomas et al. 2003) and the corresponding probability of the substitution being deleterious for protein function is high (*P*_deletirious = _0.72). PANTHER has previously been shown to accurately predict the damaging nature of Mendelian‐inherited traits that are due to a single amino acid change in the encoded protein, as is the case in our study (Thomas and Kejariwal 2004). Moreover, previous work in humans that combined PANTHER analysis with experimental validation found that a variant within a cholesterol transporter with a similarly low subPSEC score of −3.56 was highly impaired in its ability to traffic cholesterol (Brunham et al. 2005). This lends further support to the prediction that the S291F variant of the Antarctic fur seal MC1R causes a loss of function.

By comparing the S291F MC1R variant of the Antarctic fur seal with similar loss‐of‐function mutations of the receptor in other mammals, we can show that the causative mutations have multiple origins (see Fig. 2). However, many of these mutations appear to be localized within one of the seven membrane domains, suggesting that changes to the highly conserved structural components of the receptor may be particularly damaging. Additionally, a number of substitutions outside the membrane domains are known to affect coloration in, for example, mice, mammoths, and humans (Schioth et al. 1999; Hoekstra et al. 2006; Rompler et al. 2006). Many of these mutations that are not confined to transmembrane regions are either associated with a premature stop codon, as is the case for dogs and cattle (Joerg et al. 1996; Newton et al. 2000), or a frameshift mutation as in *yellow recessive* mice (Robbins et al. 1993).

There are also some similarities between the S291F substitution and loss‐of‐function MC1R variants found in other species. First, a variant associated with a similar cream‐colored phenotype in Kermode bears is situated in close vicinity to the S291F substitution within the seventh membrane domain (Fig. 2). Second, one of the substitutions implicated in the red hair phenotype of humans also lies within the same transmembrane domain, raising the possibility that this region could be a hotspot for loss‐of‐function mutations. Third, a mutation associated with a chestnut coat in domestic horses similarly leads to a serine to phenylalanine substitution, although this resides within a different transmembrane domain (Marklund et al. 1996).

Broadly speaking, *MC1R* loss‐of‐function mutations associated with distinct color morphs result in a shift of pigment deposition on the biochemical level (Majerus and Mundy 2003, Kaelin and Barsh, 2013). There are two kinds of melanin pigments produced by mammalian melanocytes, the black to brown eumelanin and the red to yellow pheomelanin. The production of these pigments is controlled by the stimulation or suppression of the MC1R, which results, respectively, in eumelanin and pheomelanin deposition (Barsh 1996). A loss or impairment of MC1R function shifts the balance of pigment production in favor of pheomelanin, which is usually expressed as yellow or red coat or hair, as seen in *recessive yellow* mice (Robbins et al. 1993) or red hair of humans (Schioth et al. 1999). This basic pigmentation is further modified in many animals, where individuals of different breeds that are homozygous for the recessive *e* allele of the *MC1R* show a range of coat colors from red through cream‐colored to white (Schmutz et al. 2002; Guibert et al. 2004; Oguro‐Okano et al. 2011). The cream coat of hypopigmented fur seals is most likely the result of a dilution of pheomelanin, as has been suggested for the hypopigmented Kermode bear and the Labrador breed of domestic dogs (Newton et al. 2000; Ritland et al. 2001). Variants of genes associated with pheomelanin dilution responsible for several different phenotypes have been identified in multiple mammal species (Mariat et al. 2003; Chintala et al. 2005). None of these, however, give rise to the cream‐colored coat observed in several dog and some cattle breeds (Guibert et al. 2004; Schmutz and Berryere 2007), which is also most similar to that of hypopigmented fur seals. It has previously been suggested that the dilution of pheomelanin in cream‐colored dogs might be due to a reduced expression of *MC1R* in melanocytes compared to dogs with a red coat (Newton et al. 2000). This reduction could be attributed to regulatory elements acting on a unique *MC1R* haplotype as found in Labradors (Newton et al. 2000), a hypothesis that is consistent with our having found a unique *MC1R* haplotype in all cream‐colored pups.

A final line of evidence pointing toward the locus in question being responsible for the cream‐colored fur seal phenotype is provided by a simple allele frequency analysis. Based on the observed frequency of the S291F substitution among wild‐type animals (0.053) and assuming HWE (the locus does not deviate significantly in wild‐type pups, *P *=* *1.00), the frequency of S291F homozygotes in the wider population can be estimated at 0.0028. This is consistent with previous estimates of the frequency of hypopigmented fur seals, which range from around one in 600–1500 (Bonner 1968) at South Georgia to one in 1700 at the South Shetland Islands (Aguayo 1978). Our estimate is slightly higher but in fact may be more accurate, as it incorporates information from randomly selected wild‐type individuals and will therefore be unaffected by observational bias. By implication, genetic analysis could provide a straightforward means of monitoring spatial and temporal variation in the frequency of hypopigmented animals, which in turn could be helpful for gaining a better understanding of global patterns of population connectivity (Bonin et al. 2013).

One potential problem with association studies such as ours is that cryptic population structure can generate spurious associations as a by‐product of genome‐wide differentiation (Xu and Shete 2005; Hoffman et al. 2014). Although this seems relatively unlikely to be an issue in studies of apparently unstructured natural populations, we do not know of any similar studies of wild animals that have tested for background genetic divergence. As a conservative measure, we therefore used our dataset of 50 microsatellite loci to test for the presence of cryptic population structure. In support of previous studies of the breeding population of fur seals at South Georgia (e.g., Hoffman et al., 2011; Stoffel et al. 2015), Bayesian cluster analysis yielded a best clustering solution of *K *=* *1. With such a large number of loci, this finding allows us to rule out the possibility that cream‐colored individuals are genetically divergent from wild‐type individuals and thus lends further support to the notion that the S291F substitution causes hypopigmentation.

Color gene polymorphisms have previously been implicated in fitness variation in a variety of vertebrate species. For example, a mutation in the pigmentation gene *agouti* is associated with the yellow obese syndrome of mice (Miltenberger et al. 1997), while in common buzzards intermediately melanized birds that are heterozygous at the *MC1R* have greater reproductive success than homozygotes (Chakarov et al. 2008) Of particular relevance to Antarctic fur seals is the recent discovery that hypopigmentation is associated via the expression of a recessive loss‐of‐function allele with inbreeding in the captive albino gorilla *Snowflake* (Prado‐Martinez et al. 2013a). As multilocus heterozygosity, a proxy for inbreeding, correlates positively with multiple fitness traits in our study population of fur seals, we therefore tested whether homozygosity at the *MC1R* could provide a marker for genome‐wide heterozygosity and thus potentially capture fitness variation. Despite our having used an unusually large panel of genetic markers, we found no significant difference in sMLH between wild‐type and cream‐colored pups. This is consistent with a recent study of common buzzards that found no association between heterozygosity at the MC1R and multilocus heterozygosity (Boerner et al. 2013). We therefore conclude that hypopigmentation does not appear to provide a phenotypic marker of reduced genome‐wide heterozygosity in fur seals and hence that cream‐colored individuals are unlikely to suffer disproportionately from the harmful effects of inbreeding depression.

A comprehensive census of the Bird Island seal population conducted in 2003 at the peak of the fur seal pupping season (Jaume Forcada, BAS, unpubl. data) points toward a similar conclusion. One in 620 pups was hypopigmented (46 of a total of 28,480 pups), one in 1560 breeding females (aged three or above) was hypopigmented (19 of a total of 29,733), and one in 1940 territorial males (aged nine or above) was hypopigmented (four of a total of 7747). With an annual survival rate of approximately 0.51 (from an independent long‐term study; BAS, unpubl. data), only one in 450 male pups born is expected to reach the age of territory tenure, and only the fittest males tend to successfully reach that age. With a total count of 46 hypopigmented pups born on the island, of which approximately half are expected to be male, assuming the same survival rate as the other pups, none would be expected to reach the age of territory tenure and be able to hold a territory. Therefore, the count of at least four hypopigmented territorial males provides circumstantial evidence that cream‐colored individuals are not necessarily less fit than wild‐type individuals.

Given that we have no evidence for hypopigmentation being associated with reduced fitness, the low frequency of the causative substitution in wild fur seal populations would appear to be attributable to founder effects and/or genetic drift. These effects may have been exacerbated by a severe bottleneck experienced by this species in the late 19th century (Hoffman et al. 2011). To explore how these processes could have influenced the frequency of the causative mutation, it would be interesting to carry out a temporal analysis, ideally comparing historical prebottleneck samples with those from the current day.

In conclusion, we used a candidate gene approach to identify a nonsynonymous mutation within the *MC1R* that is perfectly associated with hypopigmentation in a wild marine mammal population. Although all of the cream‐colored individuals are homozygous at the S291F substitution, their multilocus heterozygosity does not differ significantly from that of wild‐type individuals. This suggests that, at least in the context of inbreeding depression, hypopigmented fur seals may not be at a selective disadvantage relative to wild‐type individuals.

## Conflict of Interest

The authors declare that they have no competing interests.

## Supporting information

Raw microsatellite data are available in Table 1 in File S1. The *MC1R* sequence data are summarized in Tables S3 and S4 and are also provided in File S1. All unique *MC1R* sequences have also been submitted to Genbank (accession numbers pending).
**Figure S1.** Bayesian phylogenetic tree showing the relationship of the wild‐type Antarctic fur seal *MC1R* sequence to that of other vertebrates.
**Figure S2.** Results of the Structure analysis of the microsatellite dataset, showing average log‐likelihood values based on five replicates for each value of *K*, the hypothesized number of clusters in the data.
**Table S1.** Details of the samples used in this study.
**Table S2.** Genbank accession numbers of MC1R protein sequences used in Bayesian phylogenetic tree (Fig. S1).
**Table S3.** Alignment of DNA fragments representing *MC1R* unique haplotypes.
**Table S4**. Fisher exact test statistic and associated *P‐*value for all identified variable sites within the *MC1R* nucleotide sequence.
**Table S5.** Literature references for recessive loss of function *MC1R* mutations shown in Figure 2.
