# Supplementary material for: Born blonde: a recessive loss‐of‐function mutation in the melanocortin 1 receptor is associated with cream coat coloration in Antarctic fur seals
Source: Ecol Evol. 2016 Jul 22;6(16):5705–17. doi: 10.1002/ece3.2290 (PMC4983585; doi:10.1002/ece3.2290)
Supplement: Supplementary file 1 — Raw microsatellite data are available in Table 1 in File S1. The MC1R sequence data are summarized in Tables S3 and S4 and are also provided in File S1. All unique MC1R sequences have also been submitted to Genbank (accession numbers pending). Figure S1. Bayesian phylogenetic tree showing the relationship of the wild‐type Antarctic fur seal MC1R sequence to that of other vertebrates. Figure S2. Results of the Structure analysis of the microsatellite dataset, showing average log‐likelihood values based on five replicates for each value of K, the hypothesized number of clusters in the data. Table S1. Details of the samples used in this study. Table S2. Genbank accession numbers of MC1R protein sequences used in Bayesian phylogenetic tree (Fig. S1). Table S3. Alignment of DNA fragments representing MC1R unique haplotypes. Table S4. Fisher exact test statistic and associated P‐value for all identified variable sites within the MC1R nucleotide sequence. Table S5. Literature references for recessive loss of function MC1R mutations shown in Figure 2. [file ECE3-6-5705-s001.docx]

**Appendix**

**Supporting information legends**

**Figure S1.** Bayesian phylogenetic tree showing the relationship of the wild-type Antarctic fur seal *MC1R* sequence to that of other vertebrates. For accession numbers corresponding to the sequences used, see electronic supplementary material, table S5. Posterior probabilities are shown for all of the nodes. For illustrative purposes, the caniforms, feliforms and artiodactyls are depicted in blue, red and green respectively.

**Figure S2**. Results of the Structure analysis of the microsatellite dataset, showing average log-likelihood values based on five replicates for each value of *K*, the hypothesized number of clusters in the data.

**Table S1.** Details of the samples used in this study.

**Table S2.** Genbank accession numbers of MC1R protein sequences used in Bayesian phylogenetic tree (Fig. S1).

**Table S3**. Alignment of DNA fragments representing *MC1R* unique haplotypes. Variable sites are indicated by letters, a dot represents the same sequence as haplotype 1.

**Table S4**. Fisher exact test statistic and associated *P-*value for all identified variable sites within the *MC1R* nucleotide sequence.

**Table S5.** Literature references for recessive loss of function *MC1R* mutations shown in figure 2.

**Supplementary figure S1**

**
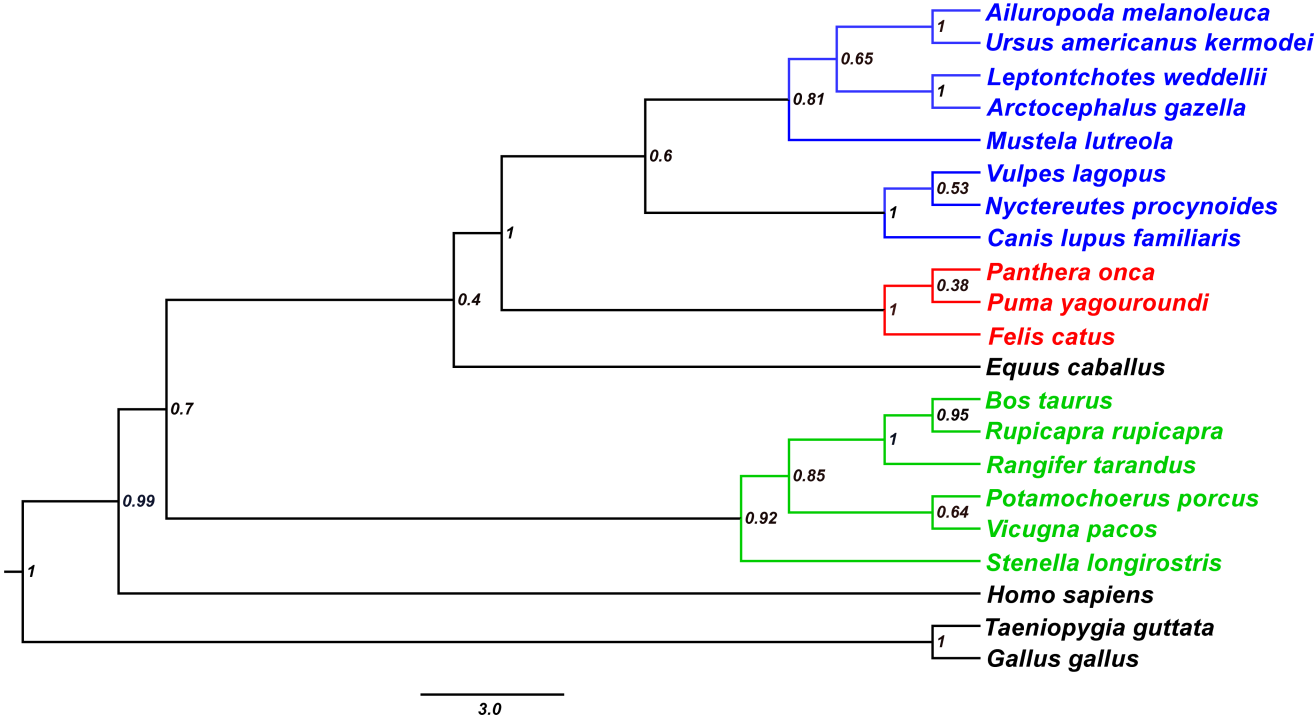
**

**Supplementary figure S2**.


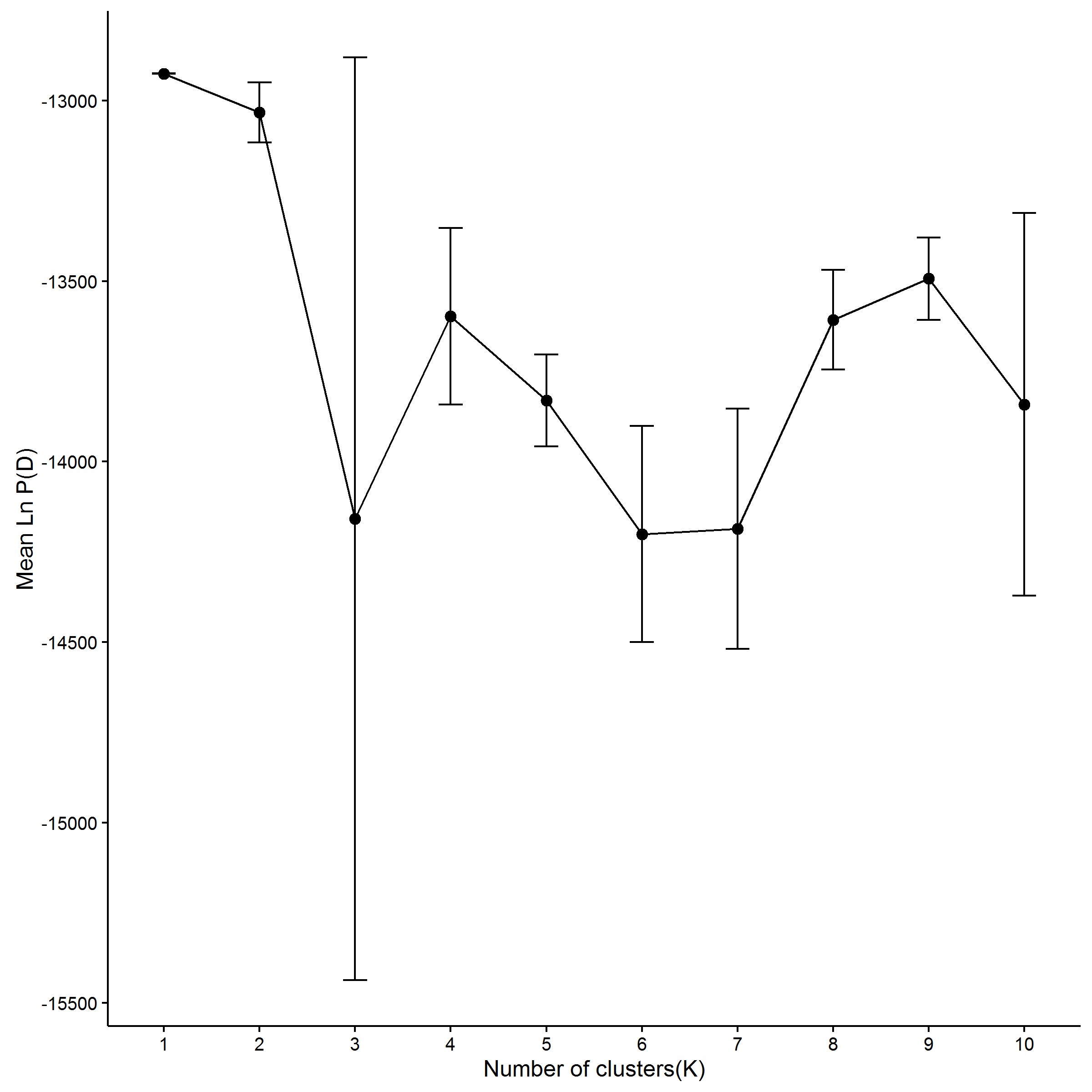


**Supplementary table S1**

| Individual ID | Life stage | Phenotype | Year of collection |
| --- | --- | --- | --- |
| 001MRAGP97 | pup | cream coloured | 1997 |
| 002MRAGP97 | pup | cream coloured | 1997 |
| 003MRAGP97 | pup | cream coloured | 1997 |
| 004MRAGP97 | pup | cream coloured | 1997 |
| W01 | pup | cream coloured | 2005 |
| W03 | pup | cream coloured | 2005 |
| W04 | pup | cream coloured | 2005 |
| W05 | pup | cream coloured | 2005 |
| W06 | pup | cream coloured | 2005 |
| W07 | pup | cream coloured | 2005 |
| W08 | pup | cream coloured | 2005 |
| W09 | pup | cream coloured | 2005 |
| W10 | pup | cream coloured | 2005 |
| W11 | pup | cream coloured | 2005 |
| W12 | pup | cream coloured | 2005 |
| W13 | pup | cream coloured | 2005 |
| W14 | pup | cream coloured | 2005 |
| W15 | pup | cream coloured | 2005 |
| W16 | pup | cream coloured | 2005 |
| W17 | pup | cream coloured | 2005 |
| AGP12065 | pup | cream coloured | 2012 |
| AGP14368 | pup | cream coloured | 2014 |
| AGP96002 | pup | wild-type | 1996 |
| AGP96008 | pup | wild-type | 1996 |
| AGP96018 | pup | wild-type | 1996 |
| AGP96020 | pup | wild-type | 1996 |
| AGP96033 | pup | wild-type | 1996 |
| AGP96036 | pup | wild-type | 1996 |
| AGP96070 | pup | wild-type | 1996 |
| AGP96081 | pup | wild-type | 1996 |
| AGP05002 | pup | wild-type | 2005 |
| AGP05003 | pup | wild-type | 2005 |
| AGP05005 | pup | wild-type | 2005 |
| AGP05006 | pup | wild-type | 2005 |
| AGP05007 | pup | wild-type | 2005 |
| AGP05008 | pup | wild-type | 2005 |
| AGP05010 | pup | wild-type | 2005 |
| AGP05011 | pup | wild-type | 2005 |
| AGP05012 | pup | wild-type | 2005 |
| AGP05013 | pup | wild-type | 2005 |
| AGP05015 | pup | wild-type | 2005 |
| AGP05016 | pup | wild-type | 2005 |
| AGP05017 | pup | wild-type | 2005 |
| AGP05018 | pup | wild-type | 2005 |
| AGP05023 | pup | wild-type | 2005 |
| AGP05025 | pup | wild-type | 2005 |
| AGP05026 | pup | wild-type | 2005 |
| AGP05027 | pup | wild-type | 2005 |
| AGP11009 | pup | wild-type | 2011 |
| AGP11014 | pup | wild-type | 2011 |
| AGP11022 | pup | wild-type | 2011 |
| AGP11026 | pup | wild-type | 2011 |
| AGP11018 | pup | wild-type | 2011 |
| AGP11032 | pup | wild-type | 2011 |
| AGP11051 | pup | wild-type | 2011 |
| AGP11041 | pup | wild-type | 2011 |
| AGP11078 | pup | wild-type | 2011 |
| AGP11065 | pup | wild-type | 2011 |
| AGP11063 | pup | wild-type | 2011 |
| AGP11079 | pup | wild-type | 2011 |
| AGP11125 | pup | wild-type | 2011 |
| AGP11144 | pup | wild-type | 2011 |
| AGP11145 | pup | wild-type | 2011 |
| AGP11130 | pup | wild-type | 2011 |
| AGP11174 | pup | wild-type | 2011 |
| AGP11151 | pup | wild-type | 2011 |
| AGP11192 | pup | wild-type | 2011 |
| AGP11185 | pup | wild-type | 2011 |
| AGP11211 | pup | wild-type | 2011 |
| AGP11200 | pup | wild-type | 2011 |
| W8913 | pup | wild-type | 2011 |
| W8914 | pup | wild-type | 2011 |
| W8915 | pup | wild-type | 2011 |
| W8916 | pup | wild-type | 2011 |
| W8917 | pup | wild-type | 2011 |
| W8918 | pup | wild-type | 2011 |
| W8919 | pup | wild-type | 2011 |
| W8920 | pup | wild-type | 2011 |
| W8921 | pup | wild-type | 2011 |
| W8922 | pup | wild-type | 2011 |
| W8923 | pup | wild-type | 2011 |
| W8924 | pup | wild-type | 2011 |
| W8925 | pup | wild-type | 2011 |
| W8926 | pup | Wild-type | 2011 |
| W8927 | pup | wild-type | 2011 |
| W8936 | pup | wild-type | 2011 |
| W8937 | pup | wild-type | 2011 |
| W8939 | pup | wild-type | 2011 |
| MRBM | adult male | cream coloured | 1996 |
| W5309/10 | adult female | cream coloured | 1997 |
| W5509/10 | adult female | cream coloured | 1997 |
| W5690/91 | mother of cream coloured pup | cream coloured | 1997 |
| Y/B132/33 | mother of cream coloured pup | wild-type | 1996 |
| Y/B134/35 | mother of cream coloured pup | wild-type | 1996 |
| Y/B142/43 | mother of cream coloured pup | wild-type | 1996 |
| Y/B144/45 | mother of cream coloured pup | wild-type | 1996 |

**Supplementary table S2**

| Species name | Accession number | Sequence length (aa) |
| --- | --- | --- |
| *Vicugna pacos* | ACL36487 | 317 |
| *Vulpes lagopus* | AHY21738.1 | 317 |
| *Felis catus* | AEE25824.1 | 317 |
| *Rupicapra rupicapra* | AGI13118.1 | 317 |
| *Gallus gallus* | AGY49276.1 | 314 |
| *Bos taurus* | NP_776533.1 | 317 |
| *Mustela lutreola* | BAE47157.2 | 317 |
| *Equus caballus* | NP_001108006.1 | 317 |
| *Homo sapiens* | EAW66676.1 | 317 |
| *Panthera onca* | AAO62414.1 | 312 |
| *Puma yagouaroundi* | AAO62416.1 | 317 |
| *Ursus americanus kamodei* | AEV46770.1 | 289 |
| *Ailuropoda melanoleuca* | XP_011232584.1 | 317 |
| *Leptonychotes weddellii* | XP_006746224.1 | 317 |
| *Nyctereutes procyonoides* | AFN70975.1 | 317 |
| *Potamochoerus porcus* | ACV60570.1 | 320 |
| *Rangifer tarandus* | CDI30159.1 | 317 |
| *Stenella longirostris* | AEB01936.1 | 317 |
| *Taeniopygia guttata* | AHM88426.1 | 305 |
| *Arctocephalus gazella* | KU935725 | 317 |
| *Canis lupus familiaris* | NP_001014304.2 | 317 |

**Supplementary table S3**

|  |
| --- |
| 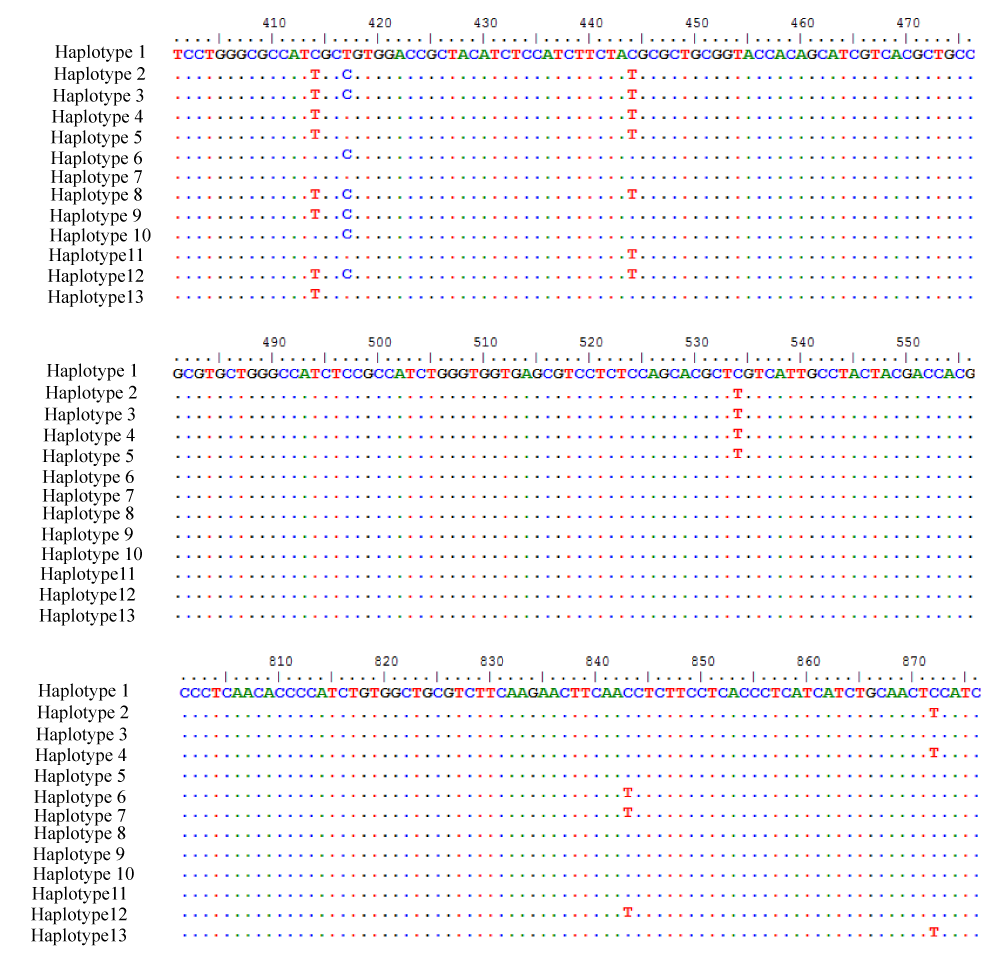 |

**Supplementary table S4**

| Nucleotide polymorphism | Fisher exact test statistic | *P*-value |
| --- | --- | --- |
| C414T | 0 | < 0.0001 |
| T417C | 0 | < 0.0001 |
| C444T | 0 | < 0.0001 |
| T534C | 0 | < 0.0001 |
| T843C | 0 | < 0.0001 |
| C872T | 0 | < 0.0001 |

**Supplementary table S5**

| Species | MC1R amino acid substitution associated with coat colour change | Reference |
| --- | --- | --- |
| Domestic dog (*Canis lupus familiaris*) | R306ter | (Newton et al., 2000) |
| Kermode bear (*Ursus americanus kermodei*) | Y298C | (Ritland et al., 2001) |
| Human (*Homo sapiens*) | R151C, R160W, D294H | (Schioth et al., 1999) |
| Domestic pig (*Sus scrofa domesticus*) | A161V, A240T | (Kijas et al., 1998) |
| Cattle (*Bos taurus*) | Y155ter | (Joerg et al., 1996) |
| Domestic horse (*Equus caballus*) | S83F | (Marklund et al., 1996) |
| Mouse (*Mus musculus*) | H183Q | (Robbins et al., 1993) |
| Beach Mouse ( Peromyscus polionotus) | R65C | (Hoekstra et al., 2006) |
| Mammoth (*Mammuthus primigenius*) | R67C | (Rompler et al., 2006) |
| Little striped whiptail lizard  (*Aspidoscelis inornata*) | T170I | (Rosenblum et al., 2010) |

**References**

HOEKSTRA, H. E., HIRSCHMANN, R. J., BUNDEY, R. A., INSEL, P. A. & CROSSLAND, J. P. 2006. A single amino acid mutation contributes to adaptive beach mouse color pattern. *Science,* 313**,** 101-104.

JOERG, H., FRIES, H. R., MEIJERINK, E. & STRANZINGER, G. F. 1996. Red coat color in Holstein cattle is associated with a deletion in the MSHR gene. *Mammalian Genome,* 7**,** 317-318.

KIJAS, J. M. H., WALES, R., TORNSTEN, A., CHARDON, P., MOLLER, M. & ANDERSSON, L. 1998. Melanocortin receptor 1 (MC1R) mutations and coat color in pigs. *Genetics,* 150**,** 1177-1185.

MARKLUND, L., MOLLER, M. J., SANDBERG, K. & ANDERSSON, L. 1996. A missense mutation in the gene for melanocyte-stimulating hormone receptor (MC1R) is associated with the chestnut coat color in horses. *Mammalian Genome,* 7**,** 895-899.

NEWTON, J. M., WILKIE, A. L., HE, L., JORDAN, S. A., METALLINOS, D. L., HOLMES, N. G., JACKSON, I. J. & BARSH, G. S. 2000. Melanocortin 1 receptor variation in the domestic dog. *Mammalian Genome,* 11**,** 24-30.

RITLAND, K., NEWTON, C. & MARSHALL, H. D. 2001. Inheritance and population structure of the white-phased "Kermode" black bear. *Current Biology,* 11**,** 1468-1472.

ROBBINS, L. S., NADEAU, J. H., JOHNSON, K. R., KELLY, M. A., ROSELLIREHFUSS, L., BAACK, E., MOUNTJOY, K. G. & CONE, R. D. 1993. PIGMENTATION PHENOTYPES OF VARIANT EXTENSION LOCUS ALLELES RESULT FROM POINT MUTATIONS THAT ALTER MSH RECEPTOR FUNCTION. *Cell,* 72**,** 827-834.

ROMPLER, H., ROHLAND, N., LALUEZA-FOX, C., WILLERSLEV, E., KUZNETSOVA, T., RABEDER, G., BERTRANPETIT, J., SCHONEBERG, T. & HOFREITER, M. 2006. Nuclear gene indicates coat-color polymorphism in mammoths. *Science,* 313**,** 62-62.

ROSENBLUM, E. B., ROMPLER, H., SCHONEBERG, T. & HOEKSTRA, H. E. 2010. Molecular and functional basis of phenotypic convergence in white lizards at White Sands. *Proceedings of the National Academy of Sciences of the United States of America,* 107**,** 2113-2117.

SCHIOTH, H. B., PHILLIPS, S. R., RUDZISH, R., BIRCH-MACHIN, M. A., WIKBERG, J. E. S. & REES, J. L. 1999. Loss of function mutations of the human melanocortin 1 receptor are common and are associated with red hair. *Biochemical and Biophysical Research Communications,* 260**,** 488-491.
